# Excessive use of WeChat, social interaction and locus of control among college students in China

**DOI:** 10.1371/journal.pone.0183633

**Published:** 2017-08-17

**Authors:** Juan Hou, Yamikani Ndasauka, Yingying Jiang, Zi Ye, Ying Wang, Lizhuang Yang, Xiaoming Li, Yongjun Zhang, Liangjun Pang, Yan Kong, Fei Xu, Xiaochu Zhang

**Affiliations:** 1 Department of Philosophy, Anhui University, Hefei, Anhui, China; 2 School of Humanities and Social Science, University of Science and Technology of China, Hefei, Anhui, China; 3 Department of Philosophy, University of Malawi; Chancellor College, Zomba, Malawi; 4 CAS Key Laboratory of Brain Function and Disease, and School of Life Science, University of Science and Technology of China, Hefei, Anhui, China; 5 Center for Biomedical Engineering, University of Science & Technology of China, Hefei, Anhui, China; 6 Anhui Mental Health Center, Hefei, Anhui, China; Nanjing Normal University, CHINA

## Abstract

In China, the number of college students using mobile phone based messaging and social networking applications like WeChat is increasing rapidly. However, there has been minimal research into the addictive nature of these applications and the psychological characteristics associate with their excessive use. There is also no published scale available for assessing excessive use of WeChat and similar applications. In the current study, we collected data from 1,245 college students in China (715 females) and developed the WeChat Excessive Use Scale (WEUS). We then assessed the relationship between excessive use of WeChat and excessive use of a social networking application-Weibo, problematic use of mobile phones, external locus of control, and social interaction skills. Our 10-item scale featured three factors, namely- “mood modification,” “salience” and ‘‘conflict”- critical factors in assessing different forms of addiction. The WEUS was found to be a reliable instrument in assessing excessive use of WeChat as it showed good internal consistency and correlated with other measures of problematic use social networking and mobile phone addiction. Our results showed that excessive users of WeChat are more likely to excessively use Weibo than they are to problematically use mobile phones. Our study also showed that greater excessive use of WeChat is associated with higher external locus of control and greater online social interaction skills. These results reveal that WeChat has unique and strong appeal among college students in China. Further, practitioners should consider dealing with malleable factors like locus of control and real life social skills in treating people with problematic messaging and social networking.

## 1 Introduction

In China, the number of college students using social networking applications like WeChat, QQ, Weibo and QZone is increasing rapidly [1]. However, the release of mobile phone based messaging applications such as WeChat is being the most popular platform with nearly the largest top 5 number of users [1, 2]. It has allowed people to socialize and stay connected longer using their mobile phones [3]. As of September 2016, there were 768 million WeChat users worldwide [4]. Despite an explosion in the popularity of WeChat, researchers have not yet investigated its addictive nature as well as psychological characteristics associated with their over-use. In fact, most existing studies have focused on effects, both positive and negative, of other computer-mediated communication (CMC) technologies, such as text messaging [5,6], email and instant messaging [7,8], social networking [9], and Weibo/microblogging [10,11]. Far less attention has been devoted to newer computer-mediated communication technologies [12,13], such as WeChat applications.

WeChat allows users to post ‘moments’ and comment on friends’ ‘moments’ like some social networking applications such as Weibo/microblogging [10,11]. It also allows text messaging, voice notes, voice calls like a mobile phone, and searching for friends within the same geographical location [4]. These features make WeChat a mobile phone based messaging as well as a social networking application. Like other social networking sites, some users of WeChat may exhibit addictive symptoms due to problematic use of this application. This problematic use may present a new and different form of Internet or behavioural addiction.

### 1.1 WeChat, mobile phones and social networking

WeChat being a mobile phone based social networking application may reflect two distinctive albeit similar addictive behaviours- mobile phone addiction and social networking addiction. On the one hand, WeChat users make constant checks on their mobile phones, a characteristic Oulasvirta, Rattenbury, Ma, and Raita [14] termed ‘checking-habit’. Further, by having different functions and activities like games, networking and messaging, WeChat, like mobile phones, is a multi-activity application/device [15]. Now, a previous study has shown that (mis)using mobile phones may lead to mobile phone addiction, which is characterized by symptoms like feeling uncomfortable and irritated when mobile phone is not accessible [16]. Mobile phone addiction is also associated with high depression [17] social extroversion, anxiety [18], insomnia [19], psychological distress [20] and loneliness [16,21,22].

On the other hand, being a social networking application, some WeChat users may largely focus their attention on social networking and exhibit addictive symptoms of social networking addiction. The use of social networking sites in general has been viewed to cause or lead to addiction [11]. In accordance with the biopsychosocial framework for the aetiology of addictions [23] and the syndrome model of addiction [24], people addicted to using social networking sites (SNSs) experience symptoms similar to those experienced by individuals who suffer from addictions to substances or other behaviours [25]. WeChat may thus be a double-edged sword warranting special inquiry into its addictive nature and psychological characteristics that may be associated with its excessive use.

### 1.2 Social interaction and locus of control

Furthermore, being a mobile phone based social networking application, the reasons for WeChat excessive use are similar to other social networking application and mobile phone addiction, associated with social interaction and locus of control. Social interaction is the essence of social networking. The advent and growth of social networking sites has allowed for more virtual interaction with diminishing real life interaction [26]. This is especially the case for socially anxious people [27]. Clinical and nonclinical cohorts of socially anxious populations report suffering from co-morbid conditions like anxiety, depression and high rates of suicide and suicidal ideations [28,29]. The presence of such co-morbid conditions amongst socially anxious people is indicative of coping difficulties. One factor likely to influence coping capability is locus of control. It is hence imperative to investigate the role of social interaction and locus of control in problematic use of messaging and social networking applications like WeChat.

Recent studies [30,31,32] have shown that different social networking sites attract different type of users and that problematic use of social networking sites is dependent on the nature and type of social networking site being investigated. As such, it is pertinent that researchers investigate problematic use of particular sites in order to understand and correctively prescribe preventative measures of this problematic use. This study intends to fill this quest by examining the excessive use of WeChat as a mobile phone based social networking application and its association with social interaction and locus of control.

#### 1.2.1 Social interaction

Social anxiety disorder is a fear of negative evaluation from others, which is greatly distressing to the individual [33]. Most people have what is considered non-clinical social anxiety, and lack social interaction skills. One of the most vulnerable groups of social anxiety is college students. Many students struggle to adjust to the social aspects of academic life including being assertive, improving self-esteem and confidence, coping with loneliness, and improving relationships [34]. Between 19% and 22% of students suffer from social anxiety [35].

With diminished social interaction skills, young people find solace in mobile phones and social networking sites, where they can engage with others without face to face interaction. Problematic use of mobile phones and social networking sites are associated with diminished real life social interaction skills [36,37]. However, more recently, Hou et al. [10] showed that use of sites like Weibo may actually enhance real life social interaction skills.

#### 1.2.2 Locus of control

The concept of locus of control, within the framework of social learning theory, depicts internal and external strength of reinforcement [38,39]. Thus, the internal and external locus of control variable integrates the behavioural paradigm of stimulus and response with cognitive theory to explain behavioural expectancies [40]. If the behaviour is viewed as determined by one’s own influence the locus of control is labelled internal and individuals with a tendency to internality are considered to be better psychologically adjusted than externals. On the contrary, if the expectancy for the behaviour is perceived as being outside one’s control it is considered ‘external’. External factors include beliefs in luck, fate, and chance.

In two studies, individuals with low social interaction skills obtained high ‘powerful others’ subscale scores on Levenson’s locus of control scale [41,42]. In contrast, Mattick and Clarke [43] reported a non-significant relationship between locus of control and social interaction skills. Further, research with college students has found that intention for playing online games as well as preference for online social interaction is greater in externals than internals [44,45]. Results from these studies suggest that internals perceive greater control over the online environment and therefore, better regulate their online behaviour. Another study found that internal locus of control was associated with greater smartphone dependency [46].

### 1.3 Current study

Despite these findings, as far as our knowledge, no study has investigated the relationship among mobile phone based messaging and social networking applications, locus of control and social anxiety. Although there have been numerous studies investigating Internet addiction and smartphone addiction [47–50], research on excessive use of mobile based social networking applications is minimal to non-existent. This study seeks to fill this gap. Thus, the present study has two objectives:—considering that there is currently no psychometric scale to assess the excessive use of WeChat, the first objective of the study is to develop a WeChat Excessive Use Scale (WEUS), assess excessive use of WeChat among college students in China, and examine the relationship between WeChat use and excessive use of mobile phones and a social networking application-Weibo. The second objective is to investigate some psychological factors of excessive use of WeChat, by examine the relationships between excessive use of WeChat and locus of control/social interaction and assess the mediating role of online social interaction between locus of control and excessive use of WeChat. We hypothesized that problematic users of WeChat would be also exhibit symptoms of social networking and mobile phone addiction. Further, we hypothesized that external locus of control and online social interaction would positively predict excessive use of WeChat use and that online social interaction would mediate the relationship between external locus of control and problematic WeChat use.

## 2 Methods and materials

### 2.1 Participants

We targeted college students in China for the study. Total number of participants was 1,245 and were recruited in and divided into two samples. Participants were recruited from 3 college campuses in Anhui province, east of China, by randomly distributed paper questionnaire to students around three university campuses. Before the comprehensive study, 10 college students (5 males and 5 females) were recruited for an index test by the same way.

### 2.2 Ethics statement

The study was approved by the Human Research Ethics Committee of the University of Science and Technology of China (USTC). All participants provided their written informed consent to participate in this study after principles expressed in the Declaration of Helsinki. We did not obtain consent from guardians of participants whose age was under 18. These young college students were considered to have comparable intelligence and ability to adult students, and able to take charge of their behaviours. Small gifts (not more than $1.5) were given as incentive to participate in the study.

### 2.3 Data collection

Planning for data collection took place before the index test. The data in samples were collected a month after the index test in a two-month interval, from November 2015 to January 2016. Paper questionnaire were randomly distributed to students around three university campuses in Anhui province. Participants were selected on the premise that they had at least one WeChat account and were college students. Those who did not complete most parts of the questionnaire (<80%) were excluded from the analyses (24/524 for Sample 1 and 22/767 for Sample 2). We used multiple imputations to replace missing values.

Sample 1 consisted of 500 college students, 240 males (48%) and 260 females (52%). The mean age was 20.7 years (*SD* = 2.1). Participants in this sample filled the 24 item WEUS and questions on characteristics of WeChat use.

Sample 2 consisted of 745 college students, 290 males (38.9%) male and 455 females (61.1%). The mean age was 19.8 years (*SD* = 1.3). Participants in this sample filled the 12 item WEUS, Mobile Phone Problem Use Scale (MPPUS), Microblog Excessive Use Scale (MEUS), Social Interaction Scale (SIS), Locus of Control Scale (LOC) and questions on characteristics of WeChat use. The MPPUS, SIS and LOC were translated from English to Chinese by two professionals. Then another pair of professionals was engaged to translate the scales back to English so as to come up with the best translation.

### 2.4 Measures

#### 2.4.1 Demographic data

Participants answered two questions concerning their gender and age.

#### 2.4.2 Characteristics of WeChat use

Participants responded to 12 questions regarding their use and perception of WeChat. Thus, we asked participants to rate their daily usage of WeChat, number of friends, activities and frequency of engaging in particular activities on WeChat. We also asked participants to rate themselves with regard to their dependence on and how important WeChat is in their lives.

#### 2.4.3 WeChat Excessive Use Scale (WEUS)

To develop our scale, we identified for components that have consistently appeared in social networking and mobile phone addiction literature [51,23,10,52]. These components are: mood modification and withdrawal symptoms (these have been considered as two sides of same coin by some scholars [53,54]; salience and relapse; conflict/Interpersonal, performance and health problems; and social comfort (a relatively new component identified by Hou et. al. in their study on excessive use of Weibo [10]).

Using this model, we formulated 24 questions (S1 File): 10 were based on responses obtained through our WeChat use interviews, 8 were adapted from Microblog excessive use scale (MEUS) and 6 were adapted from current mobile phone addiction literature (the final 10-item WEUS comprised 4 questions from our interviews and 3 questions each from MEUS and current literature). Nine questions belonged to the first component (mood modification and withdrawal symptoms). The rest of the components featured five questions each. Items were scored on a Likert-type scale ranging from “1” (“never”) to “5” (“always”).

#### 2.4.4 Microblog Excessive Use Scale (MEUS)

The scale was developed to measure excessive use of Microblogs by Hou and her fellows in 2014 [10]. In the initial study, the scale showed internal consistency and had Cronbach’s alpha of .803. MEUS has 10 items rated on a 6 point Likert scale from ‘1 = never’ to ‘6 = always’. In the current study the scale showed good internal consistency with Cronbach’s alpha of 0.911.

#### 2.4.5 Mobile Phone Problem Use Scale (MPPUS)

This scale was developed by Bianchi and Phillips in 2005 [52]. The scale has 27 questions, which are scored on a Likert-type scale ranging from 1 (“not true at all”) to 10 (“extremely true”). In the current study the Cronbach’s alpha of the scale was 0.917 showing good internal consistency.

#### 2.4.6 Social Interaction Scale (SIS)

We employed the Social Interaction Scale developed by Chen and his fellows in 2009 [55]. The Social Interaction Scale contains 24 questions divided into two parts namely- Real Life Scale (14 items) and Online Scale (10 items). The Real-Life Scale measures interpersonal communication with classmates, friends, parents, and other people in real life. The Online Scale measures individuals’ interaction with others online. The items are rated on a 4-point Likert scale from “1 = never” to “4 = always”. In our study, both the Real life and Online scales showed good internal consistency and reliability (α = 0.850 and 0.908).

#### 2.4.7 Locus of Control Scale (LOC)

We used the multidimensional locus of control scale developed by Levenson in 1981[56]. The scale has three dimensions; internal scale, which measures internal locus of control, Powerful Others Scale and Chance Scale, which measure external locus of control. In this study, we utilized the latter two scales to measure external locus of control. The scale had 16 items and were scored on a 6-point Likert scale from “1 = strongly disagree” to “6 = strongly agree”. The scale showed adequate internal consistency in our study (α = 0.796).

### 2.5 Data analysis

We analysed the data using the statistics software package SPSS 23.0 and exploratory factor analysis (EFA) was performed using Amos 23.0 software. We used data from Sample 1 for item discrimination and factor analyses. We calculated correlations between the scales using Pearson’s *r* (Pearson product-moment correlation coefficient). To test significant differences of different levels in total scores, we used the Kruskal–Wallis test (Kruskal–Wallis one-way analysis of variance) and t-tests. We also used Scheffe’s post-hoc tests to analyse significant differences between different levels. The significance level in this study was *p* < = .05.

For confirmatory factor analysis of the WEUS, we employed the robust maximum likelihood (ML) method of estimation coupled with the Satorra-Bentler scaled X2 (SB X2) correction [57] to indicate the overall goodness of fit of the model. Other fit indexes, including the comparative fit index (CFI), the normed fit index (NFI), and the root mean square error of approximation (RMSEA) were also reported to complement the SB X2-difference test [58,59].

We performed multi-group CFA to measure the moderation effect of gender on the WEUS. We also performed bootstrap analysis on our mediation model of WEUS, online social interaction and external locus of control. Further, we used a method by Lee and Preacher in 2013 [60] to calculate the difference in correlations between WEUS/MPPUS and WEUS/MEUS.

## 3 Results

### 3.1 Index test

The purpose of the index test, which involved interviews (S2 File), is to provide a reference for the development of the WEUS. It revealed interesting things about how college students use WeChat. In this test, all 10 participants said that they found WeChat a convenient way of keeping in touch with their friends, classmates and families. 7 out of 10 participants reported to have used WeChat to deal with loneliness and stress. Further, 3 out of 10 participants reported that WeChat use had affected their attention in class whilst 4 participants said they no longer felt compelled to physically meet and talk to friends because they always interact with their friends online.

### 3.2 Psychometric properties of the WEUS—Sample 1

#### 3.2.1 Item discrimination analysis

The degree of differentiation is one of the main indexes to measure the quality of the scale. We performed an inter-item correlation analysis to assess the overall agreeableness of the scale (Table 1). Item 23 was removed as it had comparatively low correlation with the total score (*r* = .304, *p <* .001).

**Table 1:** 24 items of the initial WEUS and their correlations with total score.

No	Item	Item correlation with total score
Item 1	I have used WeChat when I was bored	.555**
Item 2	I have used WeChat to relieve of loneliness and stress	.704**
Item 3	I play on WeChat as a way of relieving a bad mood like feelings of anxiety or depression	.696**
Item 4	When on WeChat, I forget about my everyday problems	.662**
Item 5	I feel happy and satisfied when I am on WeChat	.664**
Item 6	I can never spend enough time on WeChat	.655**
Item 7	I find myself saying “just a few more minutes” when browsing WeChat	.710**
Item 8	There are times when I would rather play on WeChat than go out with my friends	.605**
Item 9	I feel preoccupied with using WeChat [I think about previous WeChat activity or anticipate next opportunity to use WeChat]	.668**
Item 10	I feel the need to use WeChat with increasing amounts of time to achieve satisfaction.	.690**
Item 11	I feel depressed, moody or irritated when I can’t check messages or posts on WeChat, which goes away once I am back on WeChat	.695**
Item 12	I check my WeChat before something else that I need to do	.725**
Item 13	I feel restless, moody, depressed, or irritable when attempting to cut down or stop use of WeChat	.738**
Item 14	I have tried to spend less time on WeChat but am unable to.	.671**
Item 15	I have missed on conversations while physically with friends or family because of preoccupation with WeChat	.718**
Item 16	I have missed on some information in class or meetings because of using WeChat	.710**
Item 17	I have slept late because of preoccupation with WeChat	.702**
Item 18	My school performance or productivity has suffered because of WeChat	.668**
Item 20	I feel excited when friends comment or like my posts and pictures	.639**
Item 21	I have made new friends on WeChat which I would never have made in real life	.557**
Item 22	All my friends use WeChat	.454**
Item 23	Most people and services I know use WeChat and WeChat QR code*	.304**
Item 24	I keep in touch and know about my friends through WeChat	.595**

*: QR Code means Quick Response Code. It is a kind of black and white graphic data symbol information, which is distributed in the plane (two-dimensional direction) according to certain rules. It is a very familiar and commonly used a WeChat function, can be used for business card recognition, information acquisition, site hopping and mobile payment, etc.

** *P* < .001

#### 3.2.2 Exploratory Factor Analysis (EFA)

We use Exploratory Factor Analysis to find out the essential structure of multivariate observation variables and to process dimension reduction. Our 23-item WEUS showed good internal consistency and was subjected to an EFA. We used principle component analysis and varimax rotation. Since our scale was based on theoretical model of addiction, we computed 4 as our desirable number of factors. Values less than .400 were suppressed and factor loadings equal to and above 0.500 were considered to be indicative of items belonging to a specific factor. The four factors accounted for 62.9% of total variance.

Items 4, 9, 11, 13, 14, 15, 18 and 21 were removed because their cross-loadings were greater than .400. Thus, we remained with 15 items loading on four factors. Using the same criteria as above, we performed another EFA (details are shown in Table 2). The four factors of the second EFA accounted for 67.6% of total variance. The first factor accounted for 43.46% of the shared variance and had 4 items. We re-termed this factor “mood modification”. Cronbach’s alpha for this factor was 0.848, indicating good internal consistency.

**Table 2:** Factor loading of items from EFA of the 15-item WEUS.

	Factor 1- Mood Modification	Factor 2- Salience	Factor 3- Conflict	Factor 4- Social motivation
Item 2	**.822**	.257	.176	.128
Item 1	**.808**	-.009	.174	.117
Item 3	**.785**	.275	.179	.129
Item 5	**.612**	.325	.071	.337
Item 6	.248	**.797**	.163	.094
Item 8	.046	**.768**	.295	.042
Item 10	.155	**.758**	.318	.102
Item 7	.328	**.658**	.287	.150
Item 19	-.009	.349	**.741**	-.019
Item 16	.284	.263	**.712**	.141
Item 17	.262	.272	**.679**	.196
Item 12	.349	.336	**.516**	.261
Item 22	.047	.152	-.033	**.870**
Item 24	.381	-.075	.341	**.650**
Item 20	.341	.173	.302	**.526**

The second factor had 4 items, accounted for 11.54% of the shared variance and was re-termed “salience”. Items on relapse vanished as a result of deleting poorly loaded items above. Cronbach’s alpha for this factor was 0.847, indicating good internal consistency. The third factor had 4 items, accounted for 6.89% of the shared variance and was termed “conflict”. Cronbach’s alpha for these 4 items was 0.795, indicating good internal consistency.

The fourth factor had 3 items, accounted for 5.70% of the shared variance and was termed “social motivation and comfort”. Cronbach’s alpha for this factor was 0.669. As noted, Cronbach’s alpha for factor number 4 was less than 0.7. This indicated questionable internal consistency of the sub-scale (see [61]). As a result, we discarded factor four. We retained three factors, which consisted 12 items- 4 items in each factor. The 12-item scale showed good internal consistency with Cronbach’s alpha of 0.896.

### 3.3 Psychometric properties of the WEUS—Sample 2

#### 3.3.1 Item discrimination analysis

We performed another inter-item correlation analysis using sample 2 to assess the overall agreeableness of the scale. Item 15 was removed because its correlation with the total score (*r* = .541, *p <* .001) was less than .600 [62]. For usability of the scale [9] we also removed item 1, which had the lowest correlation with the total score (*r* = .618, *p <* .001), leaving us with a 10-Item, 3-factor scale (Table 3, S3 File).

**Table 3:** Factor structure of the 10-item WEUS.

Factor 1- Mood Modification	Factor 2- Salience	Factor 3- Conflict
Item 2	Item 6	Item 12
Item 3	Item 7	Item 16
Item 5	Item 8	Item 17
	Item 10	

#### 3.3.2 Confirmatory Factor Analysis (CFA)

We use Confirmatory Factor Analysis to test whether the relationship between a factor and the corresponding item is consistent with the theoretical relationship designed by us. Our scale and each of the three subscales had good internal consistency and reliability in the second sample; “Mood modification” (α = .818), “Salience” (α = .820), and “Conflict” (α = .735). Cronbach’s alpha for the overall WEUS was .907. Correlation between subscales of WEUS was also good (Table 4).

**Table 4:** Correlations between subscales of WEUS.

	Factor 1- Mood Modification	Factor 2- Salience	Factor 3- Conflict
**Factor 1- Mood Modification**	1	-	-
**Factor 2- Salience**	.696**	1	-
**Factor 3- Conflict**	.657**	.799**	1
**WEUS Total**	.877**	.925**	.899**

** *P* < .001

#### 3.3.3 Structure validity

**Fig 1:**
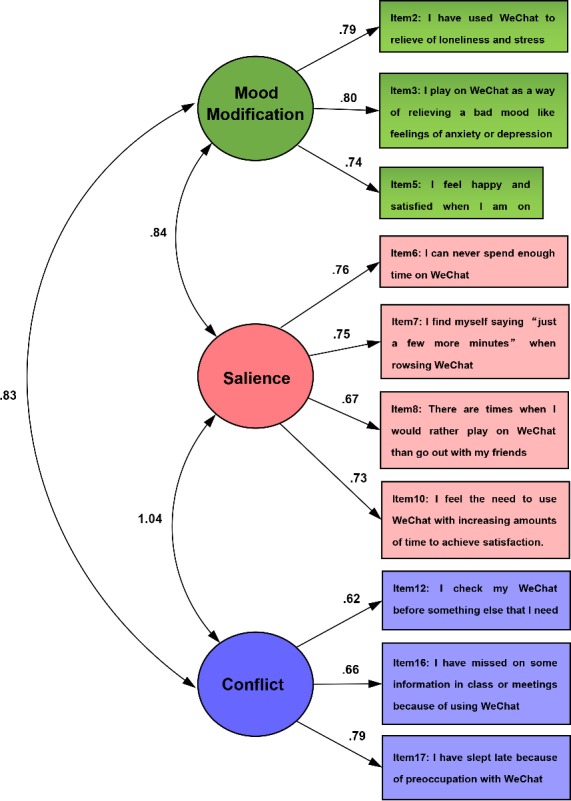
Path diagram of CFA of 10-item WEUS.

To verify the factor structure of the scale, we performed CFA and obtained the following results: Chi-square (X^2^) was 206.5; degrees of freedom was 32; comparative fit index (CFI) was .954; normed fit index (NFI) was .946; relative fit index (RFI) was .924; incremental fit index (IFI) was .954; and root mean square error of approximation (RMSEA) was .074. A proposed model is regarded to be acceptable if its values of CFI and NFI exceed 0.90, and its RMSEA value is less than 0.08 [63]. In our results, all values reached the acceptable level indicating good model fit (Fig 1).

#### 3.3.4 Criterion validity

To further assess the validity of the WEUS, we calculated the correlation between total score of WEUS and self-reports of the degree of importance of and dependence on WeChat. Our results showed significant positive correlation between WEUS total and self-report of degree of importance of (*r* = 0.525, *p* < .001) and dependence on (*r* = 0.548, *p* < .001) WeChat.

### 3.4 College students’ excessive use of WeChat—Sample 2

#### 3.4.1 Gender difference

In order to verify whether the structure model of the questionnaire is equivalent for males and females. We performed a Multi-group CFA of the WEUS with gender as a moderating factor. Constraining the measurement weights did not significantly change the model fit (*p* = .239) from the unconstrained model. Adding structural covariance constraints did not result in significant change from the unconstrained models (*p* = .131) and from the measurement weights constrained models (*p* = .145). Although CFI decreased for each model, the decreases were minimal. Overall this pattern of findings suggests that the WEUS model fitted the data equally well for both males and females (see [64]).

#### 3.4.2 Age difference

In order to investigate the differences in the excessive use of WeChat in different age groups, we divided our participants into three age groups- Group 1: 16–19 years; Group 2: 20–23 years and Group 3: 24–26 years. We analysed the difference in WEUS score among these age groups. We found significant difference in WEUS score among the three age groups (*F* = 5.319, *p* = .005). Further, we referred to Scheffe’s post-hoc tests and found significant difference between age group 1 and age group 2, the latter scoring higher than the former (MD = 1.382, *p* = .006).

#### 3.4.3 Standard of excessive use of WeChat among college students

In the absence of a standard cut-off points and clinical reports, and we can’t know the position of the individual in the general population. So, we grouped our obtained WEUS scores according to their deviation from the mean (see [65,10]). We created four groups from sample 2. Participants with a score less than the mean score (15.0) were assigned to the ‘‘non-excessive use” (NEU) group. Those whose score was between the mean score and one standard deviation (15.1 to 21.4) were assigned to the ‘‘average use” (AU) group. Participants who scored more than one standard up to 2 standard deviations from the mean (21.5 to 27.7) were assigned to the ‘‘Excessive use” (EU) group and those above 2 standard deviations (>27.7) were assigned to the “serious excessive use” group (SEU). Following this standard, 6.6% of participants fell into the SPU group, which may be associated with significant real life problems due WeChat use (detailed results are presented in Table 5).

**Table 5:** Extent of excessive use of WeChat.

Group and score	*n*	*%*
**Non-Excessive Use < 15.1**	534	71.6
**21.4 ≤ Average Use ≥ 15.1**	101	13.6
**27.7 ≤ Excessive Use > 21.4**	61	8.2
**Serious Excessive Use > 27.7**	49	6.6

#### 3.4.4 Characteristics of WeChat use and excessive use of WeChat

In Sample 2, we found significant correlation between the total duration of using WeChat and WEUS (*r* = 0.276, *p* < .001). Our analysis of variance showed significant difference among five groups of WeChat use in relation to duration. (*F* = 15.991, *p* < .001). For instance, those who had been using WeChat for less than 6 months problematically used WeChat less than those who had been using WeChat for more than 2 years and the difference was statistically significant (MD = 4.445, *p* < .001).

We undertook analysis of variance to examine the difference of frequency of sending and receiving messages on WeChat in relation to WEUS. Our results showed that there was significant difference among five levels of messaging (*F* = 30.083, *p* < .001). Further, our results showed that the more participants engaged in messaging, the higher their WEUS score (*r* = 0.413, *p* < .001).

We applied the same analysis to assess the relationship between the frequency of posting on WeChat and browsing posts on WeChat and WEUS. There was significant difference among five levels of frequency of posting on WeChat (*F* = 21.114, *p* < .001) and browsing friends’ posts on WeChat (*F* = 29.256, *p* < .001). The more frequently a participant posted on WeChat, the higher the WEUS score (*r* = 0.370, *p* < .001), and the more frequently a participant browsed friends’ posts on WeChat, the higher the WEUS score (*r* = 0.389, *p* < .001).

### 3.5 Excessive use of WeChat, excessive use of Weibo and problematic use of mobile phones

We hypothesised that excessive users of WeChat would exhibit social networking and mobile phone addiction symptoms. WEUS significantly correlated with both MEUS (*r* = 0.462, *p* < .001) and MPPUS (*r* = 0.263, *p* < .001). We calculated the difference between the two correlations and found that WEUS↔MEUS was significantly greater than WEUS↔MPPUS (*z* = 5.10, *p* < .001).

### 3.6 Excessive use of WeChat, social interaction and locus of control

Our results also showed that WEUS had significant positive correlation with online social interaction scale (*r* = 0.303, *p* < .001) as well as external locus of control scale (*r* = 0.212, *p* < .001). There was no significant association between WEUS and real life social interaction scale (*r* = 0.003, *p* = .938). Online social interaction scale also correlated with external locus of control scale (*r* = 0.178, *p* < .001).

**Fig 2:**
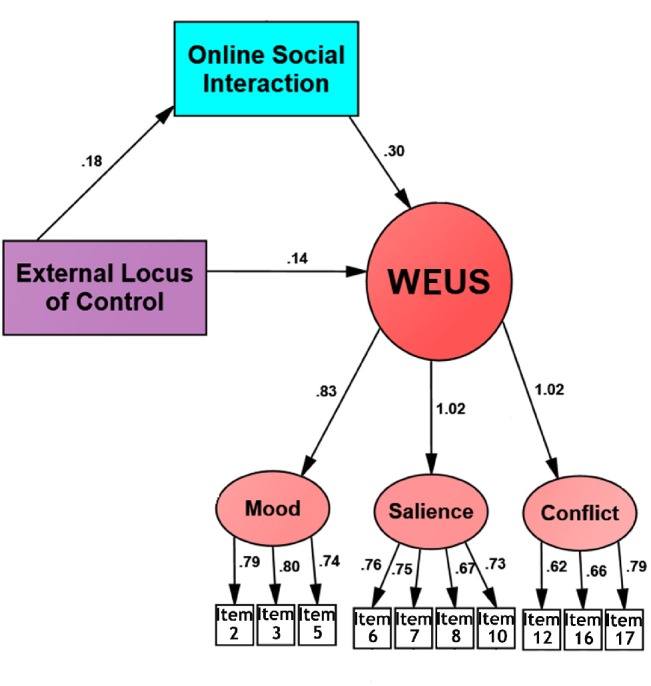
Path diagram of mediation analysis- external locus of control, online social interaction and WEUS.

Further, we performed mediation analysis among external locus of control scale, online social interaction scale and WEUS. Our results showed that online social interaction mediated the relationship between external locus of control and excessive use of WeChat (χ^2^ = 238.52, DF = 50 (*p* = .001), NFI = .940, RFI = .921, IFI = .952, CFI = .952, RMSEA = .071). The regression weights in our model indicated the existence of mediation effect (Fig 2). To ascertain this hypothesis, we performed bootstrap analysis. We used one thousand bootstrap samples and a bias corrected confidence level of 95%. Results showed a standardised direct effect of .178. With Confidence Interval of 95%, lower bound effect of .111, upper bound effect of .248 and probability of .002, we retained the null hypothesis that there was indeed mediation effect [66].

## 4 Discussion

### 4.1 Psychometric properties of the WEUS

Mobile phone messaging and social networking applications like WeChat are a relatively recent technology, being in popular use for just over a few years. Like other social networking sites, WeChat users risk problematic use. Thus, users are prone to exhibit behavioural addictions.

As previously highlighted, there is currently no published scale for assessing the excessive use of WeChat. In order to assess excessive use of WeChat, it was necessary to formulate a reliable and valid measuring instrument. In this study, we developed a scale assessing excessive use of WeChat among college students in China. We developed the scale in four stages, namely, interviews, preliminary tests, data collection, and reliability and validity data analysis. We also psychometrically analysed the scale. This process included evaluation the scales’ factor structure, its internal consistency, and its construct and criterion validity. Ultimately, we compiled a 10-item scale, composed of three factors termed “mood modification,” “salience,” and “conflict.” The scale’s reliability measured by Cronbach’s alpha was over 0.90. Thus, the WEUS exhibited good level of internal consistency as indicated by Cronbach’s alpha coefficient. The WEUS also had good construct validity as it correlated strongly with other measures of mobile phone use, namely, self-reports on participants’ regard of importance of and dependence on WeChat. The WEUS was also related to established measures of addiction—MPPUS and MEUS. Taken together, this is evidence for the construct validity of the WEUS.

### 4.2 The characteristic of excessive use of WeChat

#### 4.2.1 Gender

It is interesting to note that gender did not predict excessive use of WeChat as measured by higher scores on the WEUS. Despite conflicting results, gender has previously been found to be a function of some types of behavioural addiction. For instance, some studies have found that males more than females experience problematic use of technology and games [67–69], whilst others have suggested that women are more likely to become addicted to mobile phones and social networking than men [19,10,50]. One explanation for our results may be that the appeal of WeChat is gender neutral. That is, both males and females have embraced WeChat as a mobile messaging and social networking technology equally.

#### 4.2.2 Age

It is not surprising that higher scores on the WEUS were a function of age. What is interesting though is that we found that younger participants used WeChat less than their immediate older participants. This is inconsistent with previous studies that have shown that younger people experience more problematic use of technology than older people [67]. Our results can be explained in two ways; firstly, the age groups in our study were not so much different as both are college students. Secondly, we believe that since younger students are in junior years of their studies, and not accustomed to college pressures, they used WeChat sparingly whilst senior students did not feel the need to check on and limit their WeChat use.

### 4.3 Similarities and differences between the excessive use of WeChat and excessive use of Weibo and mobile Phones

Our results showed that excessive users of WeChat are more likely to excessively use Weibo than they are to problematically use mobile phones. This result may entail that excessive use of WeChat is an indication of social networking addiction more than it is of mobile phone addiction. Thus, WeChat and Weibo being the most popular social networking sites in China, problematic users of one also tend to problematically use the other. This result may also demonstrate the slight difference between mobile phone and social networking addictions, complimenting studies that have shown that heavier users of social networking sites exhibit different addictive symptoms from heavier users of the mobile phones [70,14].

### 4.4 Psychological factors associated with excessive use of WeChat

Our results showed that excessive use of WeChat is associated with external locus of control. Thus, people who believe that events in their lives are caused by external factors tend to over use WeChat. Externals perceive less control of their environment and hence fail to regulate their WeChat use and behaviour. This interpretation adds to the insights of [71] who surmised that the relationship an individual has with their smartphone, in our case applications, (for example level of involvement, addiction, and dependency) is a good predictor of associated negative mental health effects.

Further, our results also showed positive correlation between WEUS and online social interaction. These results were expected because previous studies have shown that people who spend much time on social networking sites also interact more online than they do in real life. WeChat users possess and develop online social interaction skills, which may later be used in real life [10]. This may explain why there was no significant association between excessive use of WeChat and real life social interaction.

Our results also showed that online social interaction mediated the relationship between external locus of control and excessive use of WeChat. Thus, externals that interact more online end up overusing WeChat. An individual with an external locus of control may prefer online interaction to real life interaction thereby problematically using social networking sites like WeChat. This may further lead to exacerbation of negative effects associated with high frequency use of social networks.

### 4.5 Study implications and suggestions for future studies

Our study makes several contributions; firstly, the current study is probably the first of this type to be undertaken within WeChat context and thus offers significant contributions to computer mediated communication literature. Furthermore, the scale developed in the study may be a useful tool for mental health practitioners in assessing excessive use of WeChat. Future studies should assess psychometric properties of the scale on other messaging applications or social networking sites.

Secondly, the results of the study reveal some critical changes within the Chinese cultural spectrum. WeChat is the first popular social networking site originating from Chinese (Eastern) culture and not from Western culture. Weibo is considered as Chinese version of Twitter whilst Renren is considered as the Chinese version of Facebook. However, the same comparison cannot be made between WeChat and WhatsApp because the two have substantial difference. Furthermore, the nature of WeChat, which allows only friends to view one’s posts, provides a greater sense of privacy than posts made on Weibo. It is not surprising that many people are shifting from Weibo to WeChat [72]. The appeal that WeChat has in China may imply that in as much as people desire collectivistic lives, a cultural element associate with Chinese culture, the desire for individuality, especially among young college students, pulls them towards seeking more privacy. This leads to ‘collectivistic individuality,’ the constant struggle to balance between the two. Studies are required to comprehensively investigate this claim.

Thirdly, results of this study have practical applications in treating excessive use of WeChat and other social networking applications. Most importantly, previous research suggests that social interaction skills and the locus of control traits are malleable, thus, they can be changed over time through careful intervention [73]. For example, many studies have found that well designed adventure programming (e.g., challenge courses and wilderness experience programs) encourages a more internal locus of control [74]. With such programming, participants discover that the environment is not unpredictable but rather can be understood and manipulated for both personal and group benefits. Further, recent studies have also suggested that good online social interaction skills can be translated into offline skills [75,76]. Considering all these along with results of the current study, it seems that psychotherapy interventions targeting locus of control and helping translate online social interaction skills into real life skills may be beneficial to individuals with difficulty controlling their use of WeChat and other addictive applications.

## 5 Limitations and conclusion

The study had some limitations that merit consideration. Firstly, data for the study were collected from participants in universities in Eastern China. The sample may not be a correct representation of colleges in China. Secondly, the study exclusively used self-report questionnaires and interviews in data collection. Self-reports are sometimes biased and not entirely reliable. Participants may have understated the extent of their WeChat use. Thirdly, there may be other psychological factors associated with excessive use of WeChat, which have not been examined in this study, such as loneliness, lack of social support and happiness, etc. Last but not the least, we didn’t compare WeChat with another popular mobile phone based social network sites like WhatsApp, so our contribution is constrained only to WeChat in China. As a result, our findings may not provide a complete picture of the extent of excessive use of WeChat in China.

In summary, the study set out to formulate a reliable and valid measuring instrument assessing excessive use of WeChat among college students in China, and investigate whether some use of WeChat, as a mobile phone based messaging and social networking application can be considered excessive use and whether such factors as social interaction skills and locus of control play a role in this excessive use. We hence developed a three-factor, 10-item scale to measure excessive use of WeChat. The scale showed good internal consistency as well as construct validity. Further, we found that excessive use of WeChat is significantly associated with online social interaction skills as well as external locus of control. Our study also showed the mediating role of online social interaction between external locus of control and excessive use of WeChat. This research may serve as a first step, indicating for clinicians and policy makers the factors that may be important when dealing with excessive use of WeChat and related mobile phone based messaging and social networking applications.

## Supporting information

S1 File WeChat questionnaire 1. (DOC)

S2 File WeChat index study. (DOC)

S3 File WeChat questionnaire 2. (DOC)
