# Supplementary material for: Excessive use of WeChat, social interaction and locus of control among college students in China
Source: PLoS One. 2017 Aug 17;12(8):e0183633. doi: 10.1371/journal.pone.0183633 (PMC5560757; doi:10.1371/journal.pone.0183633)
Supplement: S1 File — (DOC) [file pone.0183633.s001.doc]

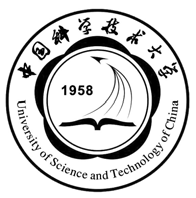
中国科学技术大学

University of Science and Technology of China

**问**

**卷**

**调**

**查**

**Study on WeChat**

**Date： / / /**

Purpose of the Study: The purpose of this study is to assess the use of WeChat among college students in China

What will be done? You will complete a survey, which will take 5 to 10 minutes. The survey includes questions about your history of using WeChat. Other survey questions will address your perceptions WeChat, the quantity and quality of your online and “real life” friendships, and your perceptions of friendship in general. We also will ask for some demographic information (e.g., age, and gender) so that we can accurately describe the general traits of the group of people who participate in the study.

Benefits of this Study: You will be contributing to knowledge about the extent to which and tendency of how people use WeChat.

Risks or discomforts: No risks or discomforts are anticipated from taking part in this study. If you feel uncomfortable with a question, you can skip that question or withdraw from the study altogether. If you decide to quit at any time before you have finished the questionnaire, your answers will NOT be recorded.

Confidentiality: Your responses will be kept completely confidential. Each participant will be assigned a participation number, and only the participant number will appear with your survey responses. Only the researchers will see your individual survey responses.

Contacts: For any inquiries, please contact Yamikani Ndasauka of University of Science and Technology of China through this email: [yami@mail.ustc.edu.cn](mailto:yami@mail.ustc.edu.cn)

Thank you for accepting to participate in the study

**Age : -------- Gender: Male Female**

**Circle the option that best describes you**

**1. How long have you been using WeChat?**

(a) 1-6 months

(b) 6 months to 1 year

(c) 1-2 years

(d) 2-3 years

(e) more than 3 years

**2. How often do you send or receive messages on WeChat?**

(a) Never

(b) Once a month

(c) Once a week

(d) Once/twice a day

(e) 5-20 times a day

(f) 21 to 50 times a day

(g) More than 50 times a day

**3. How often do you browse WeChat to post or read friends’ posts?**

(a) Never

(b) Once a month

(c) Once a week

(d) Once/twice a day

(e) 5-20 times a day

(f) 21 to 50 times a day

(g) More than 50 times a day

**4. How often do you play games on WeChat?**

(a) Never

(b) Once a month

(c) Once a week

(d) Once/twice a day

(e) 5-20 times a day

(f) 21 to 50 times a day

(g) More than 50 times a day

**5. How often do you search for and make new friends on WeChat?**

(a) Never

(b) Once a month

(c) Once a week

(d) Once/twice a day

(e) 5-20 times a day

(f) 21 to 50 times a day

(g) More than 50 times a day

**6. How often do you use WeChat to find desired products or services?**

(a) Never

(b) Once a month

(c) Once a week

(d) Once/twice a day

(e) 5-20 times a day

(f) 21 to 50 times a day

(g) More than 50 times a day

**7. How many friends do you have on WeChat**

(a) less than 10

(b) 10-20

(c) 21- 50

(d) 51-100

(e) more than 100

**8. How often do you use WeChat to advertise and sell different products or services?**

(a) Never

(b) Once a month

(c) Once a week

(d) Once/twice a day

(e) 5-20 times a day

(f) 21 to 50 times a day

(g) More than 50 times a day

**9. On a scale of 1 to 10, how much is WeChat important to you? (1 = not important and 10 = extremely important)**

1 2 3 4 5 6 7 8 9 10

**10. On a scale of 1 to 10, how much are you dependent on WeChat? (1 = not dependent and 10 = extremely dependent)**

1 2 3 4 5 6 7 8 9 10

**11. Between WeChat and Weibo, which one do you think is more private?**

(a) Weibo (b) WeChat

**12. Between WeChat and Weibo, which one do you think is more interactive?**

(a) WeChat (b) Weibo

**Using the following scale, write the number that best describes you**

**Never = 1 Occasionally = 2 Sometimes = 3 Often = 4 Always = 5**

| **No** |  | **Score** |
| --- | --- | --- |
| 1 | I have used WeChat when I was bored |  |
| 2 | I have used WeChat to relieve of loneliness and stress |  |
| 3 | I play on WeChat as a way of relieving a bad mood like feelings of anxiety or depression |  |
| 4 | When on WeChat, I forget about my everyday problems |  |
| 5 | I feel happy and satisfied when I am on WeChat |  |
| 6 | I can never spend enough time on WeChat |  |
| 7 | I find myself saying “just a few more minutes” when browsing WeChat |  |
| 8 | There are times when I would rather play on WeChat than go out with my friends |  |
| 9 | I feel preoccupied with using WeChat [I think about previous WeChat activity or anticipate next opportunity to use WeChat] |  |
| 10 | I feel the need to use WeChat with increasing amounts of time to achieve satisfaction. |  |
| 11 | I feel depressed, moody or irritated when I can’t check messages or posts on WeChat, which goes away once I am back on WeChat |  |
| 12 | I check my WeChat before something else that I need to do |  |
| 13 | I feel restless, moody, depressed, or irritable when attempting to cut down or stop use of WeChat |  |
| 14 | I have tried to spend less time on WeChat but am unable to. |  |
| 15 | I have missed on conversations while physically with friends or family because of preoccupation with WeChat |  |
| 16 | I have missed on some information in class or meetings because of using WeChat |  |
| 17 | I have slept late because of preoccupation with WeChat |  |
| 18 | My school performance or productivity has suffered because of WeChat |  |
| 20 | I feel excited when friends comment or like my posts and pictures |  |
| 21 | I have made new friends on WeChat which I would never have made in real life |  |
| 22 | All my friends use WeChat |  |
| 23 | Most people and services I know use WeChat and WeChat QR code |  |
| 24 | I keep in touch and know about my friends through WeChat |  |
