# Supplementary material for: Excessive use of WeChat, social interaction and locus of control among college students in China
Source: PLoS One. 2017 Aug 17;12(8):e0183633. doi: 10.1371/journal.pone.0183633 (PMC5560757; doi:10.1371/journal.pone.0183633)
Supplement: S2 File — (DOC) [file pone.0183633.s002.doc]

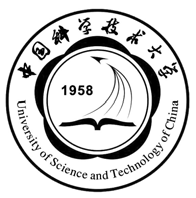
中国科学技术大学

University of Science and Technology of China

**问**

**卷**

**调**

**查**

**Study on WeChat**

**Date： / / /**

Purpose of the Study: The purpose of this study is to assess the use of WeChat among college students in China

What will be done? You will complete a survey, which will take 10 to 15 minutes. The survey includes questions about your history of using WeChat. Other survey questions will address your perceptions WeChat, the quantity and quality of your online and “real life” friendships, and your perceptions of friendship in general. We also will ask for some demographic information (e.g., age, and gender) so that we can accurately describe the general traits of the group of people who participate in the study.

Benefits of this Study: You will be contributing to knowledge about the extent to which and tendency of how people use WeChat.

Risks or discomforts: No risks or discomforts are anticipated from taking part in this study. If you feel uncomfortable with a question, you can skip that question or withdraw from the study altogether. If you decide to quit at any time before you have finished the questionnaire, your answers will NOT be recorded.

Confidentiality: Your responses will be kept completely confidential. Each participant will be assigned a participation number, and only the participant number will appear with your survey responses. Only the researchers will see your individual survey responses.

Contacts: For any inquiries, please contact Yamikani Ndasauka of University of Science and Technology of China through this email: [yami@mail.ustc.edu.cn](mailto:yami@mail.ustc.edu.cn)

Thank you for accepting to participate in the study

Age

Gender

1. How long have you been using WeChat?

2. Why do you use WeChat? (Please explain)

3. Which functions of WeChat do you use the most?

4. What are your purposes for using those functions?

5. Which function(s) of WeChat do you like the most?

6. Why do you like the function(s) (question 5)?

7. Between WeChat and Weibo, which one do you prefer? Why?

8. Between WeChat and SMS, which one do you prefer? Why?

9. What do you think are the positive changes that WeChat has brought to your life?

10. What do you think are the negative changes that WeChat has brought to your life?

11. On a scale of 1 to 10, how much is WeChat important to you? Please explain

12. On a scale of 1 to 10, how much are you dependent on WeChat? Please explain

**End of Interview**

**Thank You**
