# Supplementary material for: Excessive use of WeChat, social interaction and locus of control among college students in China
Source: PLoS One. 2017 Aug 17;12(8):e0183633. doi: 10.1371/journal.pone.0183633 (PMC5560757; doi:10.1371/journal.pone.0183633)
Supplement: S3 File — (DOC) [file pone.0183633.s003.doc]

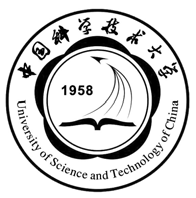
中国科学技术大学

University of Science and Technology of China

**问**

**卷**

**调**

**查**

**Study on WeChat**

**Date： / / /**

Purpose of the Study: The purpose of this study is to assess the use of WeChat among college students in China

What will be done? You will complete a survey, which will take 10 to 15 minutes. The survey includes questions about your history of using WeChat. Other survey questions will address your perceptions WeChat, the quantity and quality of your online and “real life” friendships, and your perceptions of friendship in general. We also will ask for some demographic information (e.g., age, and gender) so that we can accurately describe the general traits of the group of people who participate in the study.

Benefits of this Study: You will be contributing to knowledge about the extent to which and tendency of how people use WeChat.

Risks or discomforts: No risks or discomforts are anticipated from taking part in this study. If you feel uncomfortable with a question, you can skip that question or withdraw from the study altogether. If you decide to quit at any time before you have finished the questionnaire, your answers will NOT be recorded.

Confidentiality: Your responses will be kept completely confidential. Each participant will be assigned a participation number, and only the participant number will appear with your survey responses. Only the researchers will see your individual survey responses.

Contacts: For any inquiries, please contact Yamikani Ndasauka of University of Science and Technology of China through this email: [yami@mail.ustc.edu.cn](mailto:yami@mail.ustc.edu.cn)

Thank you for accepting to participate in the study

Demographic Data

Age

Gender: Male Female

**WeChat Usage**

1. How long have you been using WeChat?

(a) 1-6 months

(b) 6 months to 1 year

(c) 1-2 years

(d) 2-3 years

(e) more than 3 years

2. How often do you send or receive messages on WeChat?

(a) Never

(b) Once a month

(c) Once a week

(d) Once/twice a day

(e) 5-20 times a day

(f) 21 to 50 times a day

(g) More than 50 times a day

3. How often do you post on WeChat?

(a) Never

(b) Once a month

(c) Once a week

(d) Once/twice a day

(e) 5-20 times a day

(f) 21 to 50 times a day

(g) More than 50 times a day

4. How often do you browse WeChat to read friends’ posts?

(a) Never

(b) Once a month

(c) Once a week

(d) Once/twice a day

(e) 5-20 times a day

(f) 21 to 50 times a day

(g) More than 50 times a day

4. How often do you play games on WeChat?

(a) Never

(b) Once a month

(c) Once a week

(d) Once/twice a day

(e) 5-20 times a day

(f) 21 to 50 times a day

(g) More than 50 times a day

5. How often do you search for and make new friends on WeChat?

(a) Never

(b) Once a month

(c) Once a week

(d) Once/twice a day

(e) 5-20 times a day

(f) 21 to 50 times a day

(g) More than 50 times a day

6. How often do you use WeChat to find desired products or services?

(a) Never

(b) Once a month

(c) Once a week

(d) Once/twice a day

(e) 5-20 times a day

(f) 21 to 50 times a day

(g) More than 50 times a day

7. How many friends do you have on WeChat

(a) less than 10

(b) 10-20

(c) 21- 50

(d) 51-100

(e) more than 100

8. How often do you use WeChat to advertise and sell different products or services?

(a) Never

(b) Once a month

(c) Once a week

(d) Once/twice a day

(e) 5-20 times a day

(f) 21 to 50 times a day

(g) More than 50 times a day

9. On a scale of 1 to 10, how much is WeChat important to you? (1 = not important and 10 = extremely important)

1 2 3 4 5 6 7 8 9 10

10. On a scale of 1 to 10, how much are you dependent on WeChat? (1 = not dependent and 10 = extremely dependent)

1 2 3 4 5 6 7 8 9 10

11. Between WeChat and Weibo, which one do you think is more private?

(a) Weibo

(b) WeChat

12. Between WeChat and Weibo, which one do you think is more interactive?

(a) WeChat

(b) Weibo

**On a scale of 1 to 10, state how the following statements apply to your use of mobile phones (1 = not true at all and 10 = extremely true)**

| No |  | Score |
| --- | --- | --- |
| 1 | I can never spend enough time on my mobile phone. |  |
| 2 | I have used my mobile phone to make myself feel better when I was feeling down. |  |
| 3 | I find myself occupied on my mobile phone when I should be doing other things, and it causes problems. |  |
| 4 | All my friends own a mobile phone. |  |
| 5 | I have tried to hide from others how much time I spend on my mobile phone. |  |
| 6 | I lose sleep due to the time I spend on my mobile phone. |  |
| 7 | I have received mobile phone bills I could not afford to pay. |  |
| 8 | When out of range for some time, I become preoccupied with the thought of missing a call. |  |
| 9 | Sometimes, when I am on the mobile phone and I am doing other things, I get carried away with the conversation and I don’t pay attention to what I am doing. |  |
| 10 | The time I spend on the mobile phone has increased over the last 12 months. |  |
| 11 | I have used my mobile phone to talk to others when I was feeling isolated. |  |
| 12 | I have attempted to spend less time on my mobile phone but am unable to. |  |
| 13 | I find it difficult to switch off my mobile phone. |  |
| 14 | I feel anxious if I have not checked for messages or switched on my mobile phone for some time. |  |
| 15 | I have frequent dreams about the mobile phone. |  |
| 16 | My friends and family complain about my use of the mobile phone. |  |
| 17 | If I don’t have a mobile phone, my friends would find it hard to get in touch with me. |  |
| 18 | My productivity has decreased as a direct result of the time I spend on the mobile phone. |  |
| 19 | I have aches and pains that are associated with my mobile phone use. |  |
| 20 | I find myself engaged on the mobile phone for longer periods of time than intended. |  |
| 21 | There are times when I would rather use the mobile phone than deal with other more pressing issues. |  |
| 22 | I am often late for appointments because I’m engaged on the mobile phone when I shouldn’t be. |  |
| 23 | I become irritable if I have to switch off my mobile phone for meetings, dinner engagements, or at the movies. |  |
| 24 | I have been told that I spend too much time on my mobile phone. |  |
| 25 | More than once I have been in trouble because my mobile phone has gone off during a meeting, lecture, or in a theatre. |  |
| 26 | My friends don’t like it when my mobile phone is switched off. |  |
| 27 | I feel lost without my mobile phone. |  |

**On a scale of 1 to 6, state the extent to which you agree to the following statements about you (1 = strongly disagree and 6 = strongly agree)**

| No |  | Score |
| --- | --- | --- |
| 1 | Whether or not I get to be a leader depends mostly on my ability. |  |
| 2 | To a great extent my life is controlled by accidental happenings. |  |
| 3 | I feel like what happens in my life is mostly determined by powerful people. |  |
| 4 | Whether or not I get into a car accident depends mostly on how good a driver I am. |  |
| 5 | When I make plans, I am almost certain to make them work. |  |
| 6 | Of ten there is no chance of protecting my personal interests form bad luck happenings. |  |
| 7 | When I get what I want, it is usually because I’m lucky. |  |
| 8 | Although I might have good ability, I will not be given leadership responsibility without appealing to those positions of power. |  |
| 9 | How many friends I have depends on how nice a person I am. |  |
| 10 | I have often found that what is going to happen will happen. |  |
| 11 | My life is chiefly controlled by powerful others. |  |
| 12 | Whether or not I get into a car accident is mostly a matter of luck. |  |
| 13 | People like me have very little chance of protecting our personal interests when they conflict with those of strong pressure groups. |  |
| 14 | It’s not always wise for me to plan too far ahead because many things turn out to be a matter of good or bad fortune. |  |
| 15 | Getting what I want requires pleasing those people above me. |  |
| 16 | Whether or not I get to be a leader depends on whether I’m lucky enough to be in the right place at the right time. |  |
| 17 | If important people were to decide they didn’t like me, I probably wouldn’t make many friends. |  |
| 18 | I can pretty much determine what will happen in my life. |  |
| 19 | I am usually able to protect my personal interests. |  |
| 20 | Whether or not I get into a car accident depends mostly on the other driver. |  |
| 21 | When I get what I want, it’s usually because I worked hard for it. |  |
| 22 | In order to have my plans work, I make sure that they fit in with the desires of people who have power over me. |  |
| 23 | My life is determined by my own actions. |  |
| 24 | It’s chiefly a matter of fate whether or not I have a few friends or many friends. |  |

**For each item, please indicate the number to indicate the degree to which you feel the statement is characteristic or true for you in REAL LIFE**

1= Never 2= Seldom 3= Sometimes 4= Always

| No |  | Score |
| --- | --- | --- |
| 1 | I talk about my personal private matters with classmates or friends |  |
| 2 | I talk about my mood or feelings with classmates or friends |  |
| 3 | I talk about school life and school work with classmates or friends |  |
| 4 | I talk about news or current affairs with classmates or friends |  |
| 5 | I share jokes and funny moments with classmates or friends |  |
| 6 | When talking with classmates or friends, they make me feel concerned about them |  |
| 7 | I feel a sense of belonging when I talk with classmates or friends |  |
| 8 | I get useful information when I talk with classmates or friends |  |
| 9 | I talk about my secrets with my parents |  |
| 10 | I talk with my parents about what happens at school |  |
| 11 | I talk about my emotional problems with my parents |  |
| 12 | When talking with my parents, they make me feel concerned about them |  |
| 13 | I talk about my heart’s feelings or mood with my parents |  |
| 14 | I get useful information when I talk with my parents |  |

**For each item, please indicate the number to indicate the degree to which you feel the statement is characteristic or true for you on INTERNET**

1= Never 2= Seldom 3= Sometimes 4= Always

| No |  | Score |
| --- | --- | --- |
| 1 | I talk about my personal private matters with my online friends |  |
| 2 | I talk about my mood or feelings with my online friends |  |
| 3 | I talk about school life and school work with my online friends |  |
| 4 | I talk about news or current affairs with my online friends |  |
| 5 | I talk about leisure and entertainment with online friends |  |
| 6 | I talk about emotional problems with online friends |  |
| 7 | When talking with online friends, they make me feel concerned about them |  |
| 8 | I feel some company when I talk with online friends |  |
| 9 | I feel a sense of belonging when I talk with online friends |  |
| 10 | I get useful information when I talk with online friends |  |

**Please indicate the number that best describes your experience when using Weibo**

**1 = never 2 = seldom 3 = sometimes**

**4 = often 5 = usually 6 = always**

| No |  | **Score** |
| --- | --- | --- |
| 1 | How often does your school performance or productivity suffer because of twitter? |  |
| 2 | How often do you check your twitter before something else that you need to do? |  |
| 3 | How often do you find yourself saying “just a few more minutes” when browsing twitter? |  |
| 4 | How often do you feel depressed, moody or irritated when you can’t tweet, which goes away once you are back tweeting? |  |
| 5 | How often do you find yourself thinking that you would rather spend more time playing twitter than go out with your friends? |  |
| 6 | How often do you feel that you can get more followers by increasing the number of following? |  |
| 7 | How often do you feel excited when others re-tweet your tweets or comment on your tweets? |  |
| 8 | How often do you find yourself thinking of how you would try to increase the number of your followers unconsciously by all means? 2 |  |
| 9 | How often do you sleep less than 4 hours due to tweeting? |  |
| 10 | How often do you feel that your health is affected by browsing twitter? |  |

***END OF SURVEY***

***THANK YOU***
